# Supplementary material for: Clinical characteristics of 82 cases of death from COVID-19
Source: PLoS One. 2020 Jul 9;15(7):e0235458. doi: 10.1371/journal.pone.0235458 (PMC7347130; doi:10.1371/journal.pone.0235458)
Supplement: S2 Table — (DOCX) [file pone.0235458.s002.docx]

**S2 Table.** **Treatment and survival time of dead patients with COVID-19.**

| **Treatment with medications** |  |
| --- | --- |
| Intravenous of antibiotics | 82 (100) |
| Systematic corticosteroids | 29/82 (35.3) |
| Anti-virus medications | 82/82 (100) |
| **Oxygen therapy** | 82/82 (100) |
| **Mechanical ventilation** | 33/82 (40.2) |
| Invasive | 4/82 (4.8) |
| Non-invasive | 30/82 (36.6) |
| **Median time from initial symptom to diagnosis, days** | 7.0 (4.0-10.0) |
| **Median time from initial symptom to admission, days** | 10.0 (7.0-15.0) |
| **Median time from initial symptom to death, days** | 15.0 (11.0-20.0) |

Data are presented as median (IQR), or n/N (%), where N represents the total number of patients with COVID-19 with available data.
