# Supplementary material for: Clinical characteristics of 82 cases of death from COVID-19
Source: PLoS One. 2020 Jul 9;15(7):e0235458. doi: 10.1371/journal.pone.0235458 (PMC7347130; doi:10.1371/journal.pone.0235458)
Supplement: Attachment — Submitted filename: Response to Reviewers.doc [file pone.0235458.s003.doc]

Response to reviewers

Reviewer #1:

1. This article is timely given the present pandemic for COVID 19> It is a descriptive retrospective review of the epidemiological descriptions of over 80 deaths at a single hospital in Wuhan in the first month of the epidemic. as such it is an informative article. There are a few issues of note that need to be addressed:

There is no definition of "severe Illness". Results section paragraph 2 line 4 77/82 were admitted with severe illness.

**Response**: The definition has been added in the revised manuscript.

2. Line 4 of paragraph 2 of Results. Incubation time is unclear how it can be calculated. The definition in method noted this calculation to be from time of contact of transmission exposure to date to symptoms. Only 2 patients reported exposure so is incubation time calculated for only those. If so, I don't find it very informative or helpful.

**Response**: Yes, the situation at the time was too difficult to accurately trace and calculate the incubation period of each patient. Maybe, patients actually had exposure, but they did not acknowledge contact with patients confirmed with SARS-CoV-2 infection. So, we delete the data for the median incubation time (Page 11; Table 1).

3. Table 1 Comorbidity - first listing is "ALL" Does that mean ANY? Not clear what that means.

**Response**: “All” means patients having any one or more comorbidities. We have changed “All” to “Any” (Table 1).

4. Table 1 Comorbidity - please define disease of reduced immunity- is this Collagen vascular disease> is this HIV? what does this mean?

**Response**: It means abnormal blood examination for cellular immune or humoral immune indexes (Table 1).

5. Table 3 needs clarification and some editing...right now the list is just too long and difficult to find your way through--for example it notes Neutrophil count (is that a mean??) then lists % with very low and very high. This was the same for every value given. There must be an easier clearer way to present this data.

**Response**: Neutrophil count is that a median (IQR). In fact, in all Tables data are presented as median (IQR), or n/N (%), where N represents the total number of patients with COVID-19 with available data. Please see the footnotes affiliated with table 3. To present clearly, “treatment” and “survival time” in table 3 have been separated to Supporting Information part as S2 Table at the end of the manuscript.

6. Table 3 notes radiographic data in the table, but it really lists only Bilateral infiltrates at 100% of patients. This would be easier to be removed from the table and listed at the time of the statement o "severe illness on presentation - is this in fact what you meant?

**Response**: Bilateral infiltrates at 100% of patients are radiographic findings, did not equal severe illness on presentation. To present clearly, we delete this data in Table 3.

7. Table 3 what is acquired infection? nosocomial infection during admission?

**Response**: Yes, it means nosocomial infection during admission.

8. The surprise is that only 17% of patients go to MICU --- this appears first in the table 3 and then later there is a brief mention of this in the discussion. This is important and needs to move up much sooner in your paper. 83% of deaths never received Critical Care Support due to the overwhelming of your system. Only 40% had mechanical ventilation support despite 100% needing oxygen and the leading cause of death being pulmonary. This all speaks to the overwhelming of the system and needs to be highlighted. You might look to see if the patients who did make it into the MICU were any different than the ones who did not?

**Response**: The actual situation at the time was that the hospital was designated by Wuhan government as the COVID 19-designated hospital for severe cases, but the original 16 ICU beds were apparently not enough, medical staff and ventilators were extremely insufficient, and only 17% of patients go to MICU. Accordingly, there was a high mortality rate. After February 14th, as medical teams and supplies from all over the country went to and aid Wuhan, all the general wards in the hospital were transformed into ICUs and equipped with sufficient medical staff and equipment. After that, the mortality rate gradually declined. Thus, we highlighted the situation of the overwhelming of our medical system in Abstract.

9. What antiviral medications were given? Table 3 again.

**Response**: From the end of January to early February, few suitable antiviral drugs for severe patients are available. We tried LianHuaQingWen Capsule, Abidor, Ribavirin or Oseltamivir, but none seemed to have explicit effect.
